# Molecular etiological profile of atypical bacterial pathogens, viruses and coinfections among infants and children with community acquired pneumonia admitted to a national hospital in Lima, Peru

**DOI:** 10.1186/s13104-017-3000-3

**Published:** 2017-12-06

**Authors:** Juana del Valle-Mendoza, Wilmer Silva-Caso, Angela Cornejo-Tapia, Fiorella Orellana-Peralta, Eduardo Verne, Claudia Ugarte, Miguel Angel Aguilar-Luis, María del Carmen De Lama-Odría, Ronald Nazario-Fuertes, Mónica Esquivel-Vizcarra, Verónica Casabona-Ore, Pablo Weilg, Luis J. del Valle

**Affiliations:** 1 School of Medicine, Research and Innovation Centre of the Faculty of Health Sciences, Universidad Peruana de Ciencias Aplicadas, Av. San Marcos cdra. 2. Cedros de Villa, Chorrillos, Lima, Peru; 2 Instituto de Investigación Nutricional, Av. La Molina 1885, Lima 12, Peru; 3 Hospital Nacional Cayetano Heredia, Lima, Peru; 4 Barcelona Research Center for Multiscale Science and Engineering, Departament d’Enginyeria Quıímica, EEBE, Universitat Politècnica de Catalunya (UPC), Barcelona Tech, C/Eduard Maristany, 10-14, Ed. I2, 08019 Barcelona, Spain

**Keywords:** Respiratory viruses, Respiratory infection, Atypical pathogens, Community-acquired pneumonia, CAP

## Abstract

**Objective:**

The main objective of this study was to detect the presence of 14 respiratory viruses and atypical bacteria (*Mycoplasma pneumoniae*, *Chlamydia pneumoniae*), via polymerase chain reaction in patients under 18 years old hospitalized due to community-acquired pneumonia (CAP) from Lima, Peru.

**Results:**

Atypical pathogens were detected in 40% (58/146); viral etiologies in 36% (52/146) and coinfections in 19% (27/146). The most common etiological agent was *M. pneumoniae* (n = 47), followed by *C. pneumoniae* (n = 11). The most frequent respiratory viruses detected were: respiratory syncytial virus A (n = 35), influenza virus C (n = 21) and parainfluenza virus (n = 10). Viral-bacterial and bacterium-bacterium coinfections were found in 27 cases. In our study population, atypical bacteria (40%) were detected as frequently as respiratory viruses (36%). The presence of *M. pneumoniae* and *C. pneumoniae* should not be underestimated as they can be commonly isolated in Peruvian children with CAP.

**Electronic supplementary material:**

The online version of this article (10.1186/s13104-017-3000-3) contains supplementary material, which is available to authorized users.

## Introduction

Community-acquired pneumonia (CAP) is defined as an acute infection within the lungs diagnosed by clinical features and lung imaging in a previously healthy person due to an infection acquired outside of a healthcare setting [1]. This illness is the leading cause of death worldwide among children under 5 years old, representing 2 million deaths per year [2, 3]. According to the British Thoracic Society, the clinical features associated with CAP within this age group include fever, tachypnea, breathlessness, cough, wheeze or chest pain [4].

In developing countries, the etiological data from children with CAP were obtained from reports between 1980 and 1990 that mainly used serological methods [5] and also some low-level evidence descriptive studies [4, 6]. Most of the studies describing the causative agent of CAP in children are limited by the low yield of cultures, the difficulty of obtaining adequate sputum specimens and the reluctance to perform lung aspirations and bronchoalveolar lavages in this population [4].

The main objective of this study was to detect the presence of 14 respiratory viruses and atypical bacteria (*Mycoplasma pneumoniae, Chlamydia pneumoniae*) in patients under 18 years old hospitalized due to CAP from Lima, Peru.

## Main text

### Materials and methods

#### Patients and study design

A consecutive cross-sectional study was conducted in patients under 18 years of age, admitted to *Hospital Cayetano Heredia in Lima*-*Peru* with the diagnosis of community acquired pneumonia (CAP). Patients who fulfilled the selection criteria were studied from January 2009 to December 2010.

##### Inclusion criteria

Patients who were hospitalized in the pediatrics wards with the diagnosis of CAP during the study period.

##### Exclusion criteria

Patients who were diagnosed with pneumonia 48–72 h after being admitted. Patients who were admitted to the ICU service with the diagnosis of pneumonia or severe pneumonia. Patients who were transferred from other hospitals to the pediatrics wards with the diagnosis of pneumonia.

For each patient, a questionnaire with clinical and epidemiological features was completed by the physician who admitted the patient. The questionnaire applied was designed by the government for pneumonia surveillance and includes the following information: age, gender and relevant clinical information (onset, fever higher than 38 °C, cough, headache, ear pain, photophobia, conjunctival congestion, rhinorrhea, wheezing, expectoration, pharyngeal congestion, sore throat, malaise, abdominal pain, nausea, vomiting, diarrhea, lymphadenopathy, fatigue, arthralgias and myalgias).

#### Ethics statement

This study has been approved by two independent Ethics Committees from *Hospital Cayetano Heredia* and *Instituto de Investigación Nutricional*. All samples were analyzed after a written informed consent was signed by parents or children’s caregivers.

#### Samples

Nasopharyngeal samples were obtained by inserting a swab into both nostrils parallel to the palate (Mini-Tip Culture Direct, Becton-Dickinson Microbiology System, MD 21152, USA) and a second swab from the posterior pharyngeal and tonsillar areas (Viral Culturette, Becton-Dickinson Microbiology Systems, MD, USA). Both nasal and pharyngeal swabs were placed into the same tube containing viral transport medium (minimal essential medium with 2% fetal bovine serum, amphotericin B 20 μg/ml, neomycin 40 μg/ml,). Two aliquots of each fresh specimen were stored at – 20 °C to be later analyzed for respiratory viruses and atypical bacteria.

#### Reverse transcription polymerase chain reaction (RT-PCR) for the analysis of respiratory viruses

For the multiplex RT-PCR, viral genomic RNA and DNA were extracted from a total volume of 200 µl of sample by the guanidinium thiocyanate extraction method [7]. The lysis buffer included 500 molecules of the cloned amplified product used as internal control in each reaction tube and then excluded false negative results due to non-specific inhibitors or extraction failure. Two independent multiplex reverse transcription nested RT-PCR assays able to detect from 1 to 10 copies of viral genomes were performed [8, 9]. One nested RT-PCR was performed using specific primers for influenza viruses (Flu-A, Flu-B and Flu-C), respiratory syncytial viruses (RSV-A and RSV-B) and adenovirus (ADV). Another, nested RT-PCR was prepared with specific primers for detection of human parainfluenza viruses (PIV-1, PIV-2, PIV-3 and PIV-4), corona viruses (CoV-229E and CoV-OC43), human rhinoviruses (HRV), and enteroviruses (HEV). For the PCR, a single step combined RT-PCR amplification reaction, henceforth called multiplex assay 2, was performed as described previously [8, 9] (Additional file 1).

#### Polymerase chain reaction (PCR) for the analysis of *Mycoplasma pneumoniae* and *Chlamydia pneumoniae*

Polymerase chain reaction (PCR) was performed with 5 μl of template DNA, polymerase (GoTaq; Promega, Madison, Wisconsin, USA). For *M. pneumoniae*, the primers: Myco-f 5′-GAA GCT TAT GGT ACA GGT TGG-3′ and Mico-r 5-ATT ACC ATC CTT GTT GTA AGG-3′ were used; and for *C. pneumoniae*, we used primers: Clam-1f-5′-TGC ATA ACC TAC GGT GTG TT-3′ and Clam-1r 5′-TGC ATA ACC TAC GGT GTG TT-3′, Clam-2f-5′-AGT TGA GCA TAT TCG TGA TT-3′ and Clam-2r 5′-TTT ATT CCG TGT CGT CCA G-3′. The PCR consisted of initial incubation at 95 °C for 2 min, followed by 40 cycles of 95 °C for 30 s; 58 °C for 30 s, and 72 °C for 30 s; with a final extension at 72 °C for 5 min. Amplicons were detected as 275 and 225 for *M. pneumoniae* and *C. pneumoniae* respectively base pair bands after gel electrophoresis and nucleic acid staining (SybrGreen, Promega).

In each PCR assay, negative (transport medium) and positive control (cDNA) were prepared with the same procedure. Amplified products were recovered from the gel, purified (SpinPrep Gel DNA Kit; San Diego, CA) and sent for commercial sequencing (Macrogen, Korea).

#### Statistical analysis

Qualitative variables were reported as frequencies and percentages.

### Results

A total of 146 patients under 18 years old hospitalized with the diagnosis of CAP were studied. Most patients were infants under 1-year-old (81.51%) followed by children between 2 and 5 years old (11.64%). The most frequent symptoms were cough (86.96%), fever (79.45%), rhinorrhea (76.71%), and pharyngeal congestion (21.92%) (Table 1).

**Table 1:** Clinical summaries of pediatric patients with CAP

	Frequency n = cases	Prevalence (%)
Children		
Age (range)		
0–1	119	81.51
2–5	17	11.64
6–10	5	3.42
> 10	2	1.37
NR	3	2.05
Gender		
Male	87	59.59
Women	59	40.41
NR	0	0
Hospitalized	137	93.84
Sample		
Nasopharyngeal swab	145	99.32
Nasal swab	1	0.68
Nasopharyngeal aspired	0	0
Clinical symptoms		
Cough	127	86.99
Fever	116	79.45
Rhinorrhea	112	76.71
Wheezing	73	50
Expectoration	60	41.01
Pharyngeal congestion	32	21.92
Sore throat	19	13-01
Malaise	14	9.59
Vomiting	10	6.85
Diarrhea	9	6.16
Lymphadenopathy	3	2.05
Asthenia	3	2.05

Others (< 2% of cases: Ear pain, photophobia, conjunctival congestion, abdominal pain, lymphadenopathy, fatigue, myalgia)

Atypical pathogens were detected in n = 58/146 (39.72%) cases, respiratory viruses in n = 52/146 (35.62%) and coinfections in n = 27/146 (18.49%) samples; we were unable to isolate pathogens in 36 (24.66%) samples. *M. pneumonia* and RSV-A were the most common etiologies detected in 32.19% and 23.97% respectively, followed by *C. pneumoniae* (7.53%) (Table 2).

**Table 2:** Etiological diagnosis of CAP by PCR

Pathogen	Frequency (n = 146)	Prevalence (%)
Atypical pathogens (Group 1)	58	39.72
*Mycoplasma pneumoniae*	47	32.19
*Chlamydia pneumoniae*	11	7.53
Virus (Group 2)	52	35.62
RSV-A	35	23.97
Flu-A	3	2.05
Flu-C	1	0.68
CoV	1	0.68
HRV	1	0.68
PIV-1	3	2.05
PIV-2	4	2.74
PIV-4	3	2.05
HEV	1	0.68
Coinfections (Group 3)	27	18.49
*Mycoplasma pneumoniae* + RSV-A	14	9.59
*Mycoplasma pneumoniae* + HRV	1	0.68
*Mycoplasma pneumoniae* + PIV-1	1	0.68
*Mycoplasma pneumoniae* + PIV-2	1	0.68
*Mycoplasma pneumoniae* + RSV-A + HEV	1	0.68
*Mycoplasma pneumoniae* + *Chlamydia pneumoniae* + RSV-A	1	0.68
*Mycoplasma pneumoniae* + *Chlamydia pneumoniae* + Flu-A + Flu-C	1	0.68
*Chlamydia pneumoniae* + RSV-A	2	1.37
*Chlamydia pneumoniae* + CoV	1	0.68
*Chlamydia pneumoniae* + *Mycoplasma pneumoniae*	3	2.06
*Chlamydia pneumoniae* + Flu-A	1	0.68
Negative samples	36	24.66

Coinfections were detected in 27 cases (18.49%), and the most frequent association corresponded to *M. pneumoniae* with VRS-A (9.59%). No viral-viral associations were observed (Table 2).

**Fig. 1:**
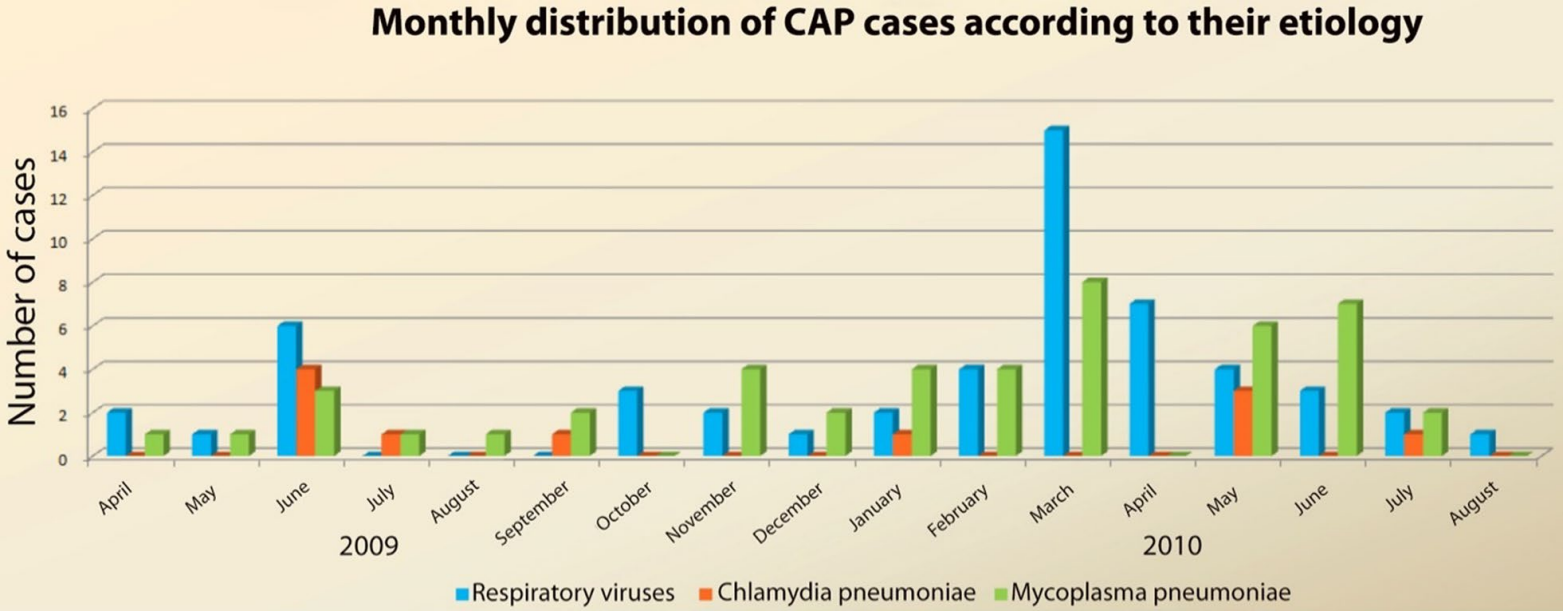
Community acquired pneumonia seasonal distribution

A monthly distribution of the CAP cases was analyzed according to their etiologies during the study period. An even distribution of infections with *C. pneumoniae* were observed across the year and a relative increase of *M. pneumoniae* was observed from March to June. An isolated peak of respiratory viruses was detected during March being RSV-A the most common isolated virus (Fig. 1).

### Discussion

Establishing the etiology of CAP in children can be challenging in developing countries due to many factors including: the difficulty to obtain adequate samples, the invasive characteristic of specific diagnostic tests and the unavailability of reliable diagnostic methods in the primary care setting. Without a sensitive and specific diagnostic method, physicians have to rely on clinical criteria based on signs and symptoms and epidemiological information of CAP to determine the possible causative agent and provide the patient with the proper treatment [2, 4, 6, 10].

Multiple studies have previously reported that respiratory viruses are the leading cause of community acquired pneumonia in children and can be detected in more than 50% of the cases [6, 11]. However, this results may vary between studies due to the differences in seasonal patterns observed in distinct areas [4, 6, 10, 12]. In our study population, atypical bacteria were slightly more frequently detected (39.73%) than respiratory viruses (35.62%).

In the group of patients with pneumonia caused by atypical pathogens, *M. pneumoniae* was the predominant microorganism and was detected in 32.19% of the samples. This finding correlates with some previous studies that have detected *M. pneumoniae* in up to 36% of children with community acquired pneumonia [4, 13]. Moreover, we observed a similar *M. pneumoniae* predominance in a previous study we conducted in children with acute respiratory illness (ARI) around the same study period. We found that in children with ARI, *M. pneumoniae* was present in up to 25% (170/675) of samples and *C. pneumoniae* in 10% (71/65) [14].

The most common pathogen isolated within the group of patients with viral pneumonia was RSV type A (23.97%), followed by Parainfluenza 2 (2.74%). Other studies have reported a similar distribution of viral etiologies in children with CAP [4, 7, 11]. However, seasonal pattern variations and viral outbreaks can considerably alter the prevalence of certain viruses between surveillance studies, especially for RSV and influenza virus [12].

In our series, we observed Chlamydia pneumonia infections evenly distributed through the year, whereas a relative increase of *M. pneumoniae* was observed from March to June. However, no clear seasonal pattern can be concluded for both atypical bacteria or respiratory viruses during our study period, probably due to the limited number of cases. Nevertheless, our study demonstrates the constant presence of atypical bacteria throughout the year in patients with CAP.

In recent years, there has been an increasing interest regarding the association between bacteria and viruses in the pathogenesis of pneumonia. Studies have shown patients that had a viral infection followed by a secondary bacterial lower respiratory infection, had a higher morbidity and mortality [15, 16]. Coinfections between bacterial and viral isolates have been detected in up to 45% of pediatric patients with CAP; and the most common association has been reported to be between *Streptococcus pneumoniae* and respiratory viruses [16]. However, *M. pneumoniae* has also been described as a bacterium commonly isolated in sputum samples from young children with coinfections. Moreover, it has been proposed that patients infected with *M. pneumoniae* may be more susceptible to other infectious pathogens [17]. In our study, coinfections between *M. pneumoniae* and other microorganisms were observed in 15.73% of the samples, and RSV was the most frequent co-infective agent present in 9.59% of samples.

Several studies have demonstrated that the detection of viruses in children with CAP has been underestimated, primarily due to limited diagnostic methods and difficult sample collection [4, 15]. In this study, Nested RT-PCR was used to simultaneously detect a wide variety of viruses with a high sensitivity [8]. Furthermore, a rapid extraction method of genomic material was employed, allowing a more efficient recognition of viral RNA and even bacterial DNA.

In conclusion, our study revealed that both atypical bacteria and respiratory viruses are among the most frequent agents detected in children with CAP from Lima, Peru. The incorporation of highly sensitive and specific molecular techniques, such as RT-PCR [4], should be considered in order to achieve an accurate etiological diagnosis and therapeutic management, avoiding the empirical use of antibiotic therapy, particularly in children with pneumonia of viral etiology. In addition, an increase in macrolide resistance has been observed worldwide among CAP patients infected with *S. pneumoniae* and *M. pneumoniae*. This highlights the importance of a precise etiological diagnosis during the management of CAP in children [18].

A timely pathogen identification can prevent nosocomial spread of the disease and provide epidemiological information to healthcare networks [19], as well as provide key data to reduce the inappropriate use of antibiotics [20]. Antibiotic choice for CAP can vary widely across practices and an increasing use of broad-spectrum antibiotics have been observed by clinicians at suburban practices. In addition, factors not related to the microbiologic etiology such as age, previous antibiotic receipt or type of insurance are common arbitrary criteria used for antibiotic choice increasing the risk for drug resistance [21]. Further investigations should be conducted in Peru to have a better understanding of the role of atypical agents in CAP and the risks for antibiotic resistance.

#### Limitations

Our results have shown that RT-PCR is a more efficient diagnostic technique since it can detect multiple viruses that are not recognized by conventional methods. Nevertheless, despite the improvement in the etiological diagnosis of CAP in children, we could not identify an etiology in a significant proportion of patients. In those cases, *S. pneumoniae* could be the causative pathogen as it is the main cause of pneumonia in most age groups. However, we cannot rule out the presence of other etiological agents.

## Additional file

**Additional file 1.** Primers for Influenza Virus (Flu), respiratory syncytial virus (RSV), Human Parainfluenza Viruses (Parainf.), Coronaviruses, Enteroviruses (Enterov.), and Rhinoviruses (Rhinov.) Used in the First Round Multiplex RT-PCR and in the Following Nested PCR.
