# Supplementary material for: Molecular etiological profile of atypical bacterial pathogens, viruses and coinfections among infants and children with community acquired pneumonia admitted to a national hospital in Lima, Peru
Source: BMC Res Notes. 2017 Dec 6;10:688. doi: 10.1186/s13104-017-3000-3 (PMC5718007; doi:10.1186/s13104-017-3000-3)
Supplement: Supplementary file 1 — Additional file 1. Primers for Influenza Virus (Flu), respiratory syncytial virus (RSV), Human Parainfluenza Viruses (Parainf.), Coronaviruses, Enteroviruses (Enterov.), and Rhinoviruses (Rhinov.) Used in the First Round Multiplex RT-PCR and in the Following Nested PCR. [file 13104_2017_3000_MOESM1_ESM.docx]

**Additional file.** Primers for Influenza Virus (Flu), respiratory syncytial virus (RSV), Human Parainfluenza Viruses (Parainf.), Coronaviruses, Enteroviruses (Enterov.), and Rhinoviruses (Rhinov.) Used in the First Round Multiplex RT-PCR and in the Following Nested PCR.

| **Amplification steps and primer** | **Sequence (5´🡪 3´)** | **Gene position** | **Amplicon**  **size (bp)** |
| --- | --- | --- | --- |
| RT-PCR^a^ |  |  |  |
| FluAC1  FluB1 FluABC2 | GAACTCRTYCYWWATSWCAAWGRRGAAAT  ACAGAGATAAAGAAGAGCGTCTACAA  ATKGCGCWYRAYAMWCTYARRTCTTCAWAIGC | (A) 319–347  (C) 346–374  217–242  (A) 1040–1009  (B) 1208–1177  (C) 1084–1053 | (A) 721  (B) 991  (C) 738 |
| RSVAB1  RSVAB2 | ATGGAGYTGCYRATCCWCARRRCAARTGCAAT  AGGTGTWGTTACACCTGCATTRACACTRAATTC | 1–31  737–705 | 737 |
| 1-PIV13  2-PIV13  1-PIV2  1-PIV4  2-PIV24 | AGGWTGYSMRGATATAGGRAARTCATA  CTWGTATATATRTAGATCTTKTTRCCTAGT  TAATTCCTCTTAAAATTGACAGTATCGA  ATCCAGARRGACGTCACATCAACTCAT  TRAGRCCMCCATAYAMRGGAAATA | Parainf. 1 (641-667)  Parainf. 3 (635-661)  Parainf. 1 (1277-1248)  Parainf. 3 (1270-1241)  Parainf. 2 (259-286)  Parainf. 4 (107-81)  Parainf. 2 (942-919)  Parainf. 4 (963-940) | Parainf. 1 (635)  Parainf. 3 (635)  Parainf. 2 (683)  Parainf. 4AB (1070) |
| 1-HcoV  2-HcoV | TGTGCCATAGARGAYWTACTTTTT  AACCGCTTKYACCAKCAAYGCACA | 229E (2068-2090)  OC43 (2727-2750)  229E (2919-2896)  OC43 (3533-3511) | 229E (851)  OC43 (806) |
| 1-EV/RV  2-EV/RV | CTCCGGCCCCTGAATRYGGCTAA  TCIGGIARYTTCCASYACCAICC | Enterov. 445-467  Rhinov. 1200-1178 | Enterov. (755)  Rhinov. (639) |
| Nested^b^ |  |  |  |
| FluAB3  FluC3  FluAC4  FluB4 | GATCAAGTGAKMGRRAGYMGRAAYCCAGG  AAATTGGAATTTGTTCCTTTCAAGGGACA  TCTTCAWATGCARSWSMAWKGCATGCCATC  CTTAATATGGAAACAGGTGTTGCCATATT | (A) 718–746  (B) 892–920  952–980  (A) 1019–990  (C) 1063–1034  1118–1090 | (A) 301  (B) 226  (C) 111 |
| RSVA3  RSVA4  RSVB3  RSVB4 | TTATACACTCAACAATRCCAAAAAWACC  AAATTCCCTGGTAATCTCTAGTAGTCTGT  ATCTTCCTAACTCTTGCTRTTAATGCATTG  GATGCGACAGCTCTGTTGATTTACTATG | 347–374  710–682  30–59  641–614 | (A) 363  (B) 611 |
| 3-PIV13  4-PIV1  4-PIV3  3-PIV24  4-PIV2  4-PIV4 | ACGACAAYAGGAARTCATGYTCT  GACAACAATCTTTGGCCTATCAGATA  GAGTTGACCATCCTYCTRTCTGAAAAC  CYMAYGGRTGYAYTMGAATWCCATCATT  GCTAGATCAGTTGTGGCATAATCT  TGACTATRCTCGACYTTRAAATAAGG | Parainf. 1 (754-776)  Parainf. 3 (748-770)  Parainf. 1 (1193-1168)  Parainf. 3 (1138-1112)  Parainf. 2 (487-514)  Parainf. 4 (509-536)  Parainf. 2 784-761  Parainf. 4 683-358 | Parainf. 1 (439)  Parainf. 3 (390)  Parainf. 2 (297)  Parainf. 4AB (174) |
| 3-HcoV  4-HcoV | TTGTGCGCAATGTTATAAWGGYAT  GATAATRTGAGTRCCATTWCCACA | 229E (2174–2197)  OC43 (2831-2854)  229E (2804–2781) | 229E (630)  OC43 (587) |
| 3-EV/RV  4-EV/RV | ACCRASTACTTTGGGTRWCCGTG  CTGTGTTGAWACYTGAGCICCCA | Enterov. 536–559  Rhinov. 762–743 | Enterov. (226)  Rhinov. (110) |

^a^1, forward; 2, reverse in first-round RT-PCR.

^b^3, forward; 4, reverse in nested PCR.

Coiras MT, Pérez-Breña P, García ML, Casas I. [Simultaneous detection of influenza A, B, and C viruses, respiratory syncytial virus, and adenoviruses in clinical samples by multiplex reverse transcription nested-PCR assay.](http://www.ncbi.nlm.nih.gov/pubmed/12436489) J Med Virol. 2003 Jan; 69(1):132-44.

Coiras MT, Aguilar JC, García ML, Casas I, Pérez-Breña P. Simultaneous detection of fourteen respiratory viruses in clinical specimens by two multiplex reverse transcription nested-PCR assays. J Virol. 2004 Mar; 72 (3):484-95.
